# Dangers of Sea Bathing

**Published:** 1883-10

**Authors:** 


### DANGERS OF SEA BATHING.

SEA bathing is attended by other dangers besides that of drowning. Many persons have had their hearing impaired from the effects of having the salt water enter the ears, and there have been fatal ulcerations in the head and nasal passages from the same cause. Sea water, near the shore, holds in suspense particles of fine sand and other débris, portions of which are apt to be carried into the ears, mouth and nose with the water and find lodgment there, particularly with persons who have catarrhal affections. It is safer, therefore, to avoid getting sea water in the ears ; it is often impossible to remove these grains of fine sand from the ears and nasal passages, for they become imbedded in the lining membranes, where they are indestructible and act as permanent irritants.

PURIFYING WATER.—Water containing vegetable matter may be purified by dropping in a gallon of the water 2 or 3 drops of muriated tincture of iron. In the course of an hour or two the iron will carry all the organic matter to the bottom of the vessel, leaving the water pure and wholesome. This will not purify water that contains noxious gases, such as come from drains and cesspools.

THE RUSSIAN EMPIRE.—The Russian Empire consists of thirty different races of people. Life there is not individual, but collective. There are but two cities, Moscow and St. Petersburg ; the remaining towns are viewed but as accidents. In other countries the urban population constitutes one-third of the whole, in Russia but one-tenth. St. Petersburg and Moscow are the only cities, perhaps, in the world whose inhabitants are part peasants. The workpeople in the factories of these cities are engaged on the condition that they will be allowed vacation to sow their fields and reap their harvests.

